# Management of insomnia evaluated by the Psychiatry Interconsultation in a patient with a comorbid medical condition

**DOI:** 10.1192/j.eurpsy.2025.2328

**Published:** 2025-08-26

**Authors:** A. Blanco Barrón, M. T. González Salvador, A. Castiglioni García-Diego, M. Magariños López

**Affiliations:** 1 Psychiatry, Hospital Puerta de Hierro, Madrid, Spain

## Abstract

**Introduction:**

Insomnia, affecting about one-third of adults and worsening with age, impacts individual’s health, social life, and occupational functioning. Therefore, untreated insomnia can lead to depression. Although it can appear as an independent symptom, it most often presents as a comorbid disorder.

This paper discusses the case of a 71-year-old man with acute necrotizing pancreatitis and history of multiple admissions for recurrent abdominal pain, was assessed by Psychiatry for a possible adaptive disorder. He was diagnosed with persistent insomnia linked to his medical condition and secondary low mood. Sleep hygiene and various medications were recommended but proved ineffective. Eventually, an orexin antagonist, daridorexant, was prescribed.

**Objectives:**

The aim of this work is to orient, within the wide range of psychopharmacology available for the treatment of insomnia, the effectiveness and advantages of the use of daridorexant in patients with comorbid medical pathology.

**Methods:**

To evaluate the efficacy of the drug in improving the quantity and quality of sleep, and the diurnal impact of insomnia, the Athens scale, consisting of 8 items, was used. It has been completed with a sleep diary that provides specific information on sleep. The results obtained were compared with those published by means of a literature search in PubMed.

Permission is requested from the patient to present this case anonymously.

**Results:**

After 30 days of treatment with daridorexant, the Athens Scale score decreased, with a perceived improvement in nocturnal rest (quantity and quality of sleep) and daytime impact of insomnia, with good tolerance and no side effects.

**Conclusions:**

The pharmacological treatment of insomnia has undergone important advances in the last two decades. The treatment of insomnia is multidisciplinary and will depend on its etiology. There seems to be no single, first-choice pharmacological treatment for insomnia, which is why the options are varied and wide-ranging.

The management of this disorder seeks two fundamental objectives: to improve the quality of sleep and to improve daytime symptoms. Both are improved in this patient with the help of daridorexant. The review of the available literature supports the observed case, being daridorexant a safe and effective option for the treatment of insomnia. It is worth mentioning that in Spain, daridorexant has been approved in September 2023, so the clinical experience at present is scarce.

In our patient the drug has been well tolerated, with no reported side effects or variations in analytical parameters. With respect to insomnia, anxious and negative expectations regarding sleep, concern about the potential consequences of not sleeping enough or not sleeping well have decreased and, in short, the quality of sleep, functionality during the day, and even mood have improved in general terms.

**Disclosure of Interest:**

None Declared

